# Exploring the association between precipitation and hospital admission for mental disorders in Switzerland between 2009 and 2019

**DOI:** 10.1371/journal.pone.0283200

**Published:** 2023-04-24

**Authors:** Sujung Lee, Coral Salvador, Alexandre Tuel, Ana Maria Vicedo-Cabrera

**Affiliations:** 1 Institute of Social and Preventive Medicine, University of Bern, Bern, Switzerland; 2 Oeschger Center for Climate Change Research, University of Bern, Bern, Switzerland; 3 Environmental Physics Laboratory (EPhysLab), Centro de Investigación Mariña, Universidade de Vigo, Ourense, Spain; 4 Institute of Geography, University of Bern, Bern, Switzerland; University of Asia Pacific, BANGLADESH

## Abstract

While several studies proved the relationship between increasing temperatures and poor mental health, limited evidence exists on the effect of other weather factors, such as precipitation. This study assessed the impact of precipitation on hospital admissions for mental disorders in Switzerland between 2009–2019. We defined different precipitation events based on the duration (daily precipitation ≥1mm for 2, 3, or 4 days; PP.2/PP.3/PP.4) and intensity (≥90th percentile for 2 consecutive days; PEP90.2). First, we conducted aggregated time-stratified case-crossover analysis in eight main Swiss cities with distributed lag models to assess the association up to 3 days after the exposure. Then, we pooled the estimates in each city using a multivariate random effects meta-analysis for all hospital admissions and by subgroups (sex, age, diagnosis). Evidence of an association between precipitation and hospital admission for mental disorders was not found in Switzerland (PP.2: 1.003[0.978–1.029]; PP.3: 1.005[0.985–1.026]; PP.4: 0.994[0.960–1.030]; PEP90.2: 1.000[0.953–1.050]). Although the results were highly uncertain, we found an indication of increasing risks of hospital admission with increasing intensity of precipitation in warmer seasons (PP.2: 1.001[0.971–1.032] vs PEP90.2: 1.014[0.955–1.078]), while the risks of hospital admission slightly increased by the duration in colder season (PP.2: 1.009[0.981–1.039]; PP.3: 1.008[0.980–1.036]; PP.4: 1.017[0.956–1.081]). Overall, risks tend to be higher in people aged < 65 years. Duration of the events may influence more than intensity in females, while opposite patterns were observed in males. Risks tended to be larger but still uncertain for schizophrenia, mood disorders, and adult personality disorders. An indication of a negative association was found in neurotic disorders and null risks in the remaining groups. Although our findings did not show a clear association between precipitation and mental disorders, further research is required to clarify the role of precipitation and the potential implications of climate change and extreme precipitation events on mental health.

## Introduction

The Intergovernmental Panel on Climate Change (IPCC) Sixth Assessment Report (AR6), *Climate Change 2022*: *Impacts*, *Adaptation and Vulnerability*, states that climate change has an adverse impact on mental health, and it is expected to be exacerbated in the future with *very high confidence* [1]. Climate and weather extremes impose complex and compound mental health risks in direct and indirect ways. In general, the direct impact of climate change on mental health includes stress- or trauma-related disorders triggered by acute weather events such as cyclones or floods [2]. On the other hand, indirect effects are caused by consequential environmental risk, economic losses, or displacement due to the loss of habitable land [3].

Several studies found that both the frequency and intensity of heavy precipitation are rising in Switzerland [4,5]. Schmidli and Frei [6] showed significant trends for heavy precipitation in the winter and fall for the northern, western, and eastern regions of Switzerland. Furthermore, Froidevaux et al. [7] showed that widespread heavy precipitation is followed by floods over much of Switzerland except for the high Alpine region. Several studies also demonstrated that persistent extreme precipitation events are connected to floods [7–9] and high river discharge events [10].

Recently much attention has been paid to the psychological impacts of climate change. While many studies focused on the seasonal patterns of psychological outcomes [11–14] and/or their association with high ambient temperature [15–17] and heatwaves [16,18], limited evidence exists about the impacts of other weather factors, such as precipitation, on mental health. Several studies have found an association between precipitation or flood and mental disorders. For example, Graham et al. [19] proved that populations experiencing flood- or storm-related damages are more vulnerable to common mental disorders. Other studies suggested that changes in precipitation levels owing to climate change possibly affect food security and food insufficiency and cause socio-economic disruptions, which can be indirectly linked to increasing mental health problems [1,20–23]. Additionally, other studies found direct effects of heavy precipitation and mental health outcomes. For example, Wei et al. [24] found an association between flood and hospital admissions for schizophrenia. However, previous studies pointed out that it is necessary to differentiate the types of precipitation events and assess if there is a different impact on mental disorders [25,26]. Floods are generally caused by a mix of processes [27], so even if the studies include precipitation as an exposure variable, most did not consider precipitation events’ different characteristics, such as short-duration extreme rain, thunderstorms, or persistent moderate rain. For example, one study assessed the impact of extreme precipitation on hospital admissions for schizophrenia [28]. However, this study only identified single extreme precipitation events, not considering consecutive precipitation events, which can increase the occurrence of floods.

Hwong et al. [3] described the difficulty of assessing mental health due to its regional and cultural differences in definition and acknowledgments. Furthermore, assessing the impact of climate change on mental health is challenging as the developmental factor of mental disorders after a disaster is not equally distributed [29]. Several studies have demonstrated that due to the differential exposure, socioeconomic status, and capacity for adaptation, the psychological impact of climate change could be intensified in vulnerable individuals or communities [30–33]. In the case of Switzerland, micro-cultural differences in health context exist between cities because of unique situations; German-, French- and Italian-speaking people are united within one country [34]. Also, in the case of climatic situations, Switzerland has a different geographical distribution of the intensity and frequency of heavy precipitation [4,35]. Thus, it is necessary to assess the relationship between precipitation and hospital admissions due to mental disorders on a nationwide scale but also a local scale.

Considering increasing trends in heavy precipitation and limited evidence on its psychological impact, here we assessed the association between precipitation and the risk of hospital admission due to mental disorders in eight main Swiss cities between 2009 and 2019. We conducted a comprehensive assessment across population subgroups by age and sex and diagnosis on a city scale. We also explored the potential seasonal patterns of the effects and the robustness of our findings by conducting a sensitivity analysis.

## Data

### Hospital admission data

**Fig 1:**
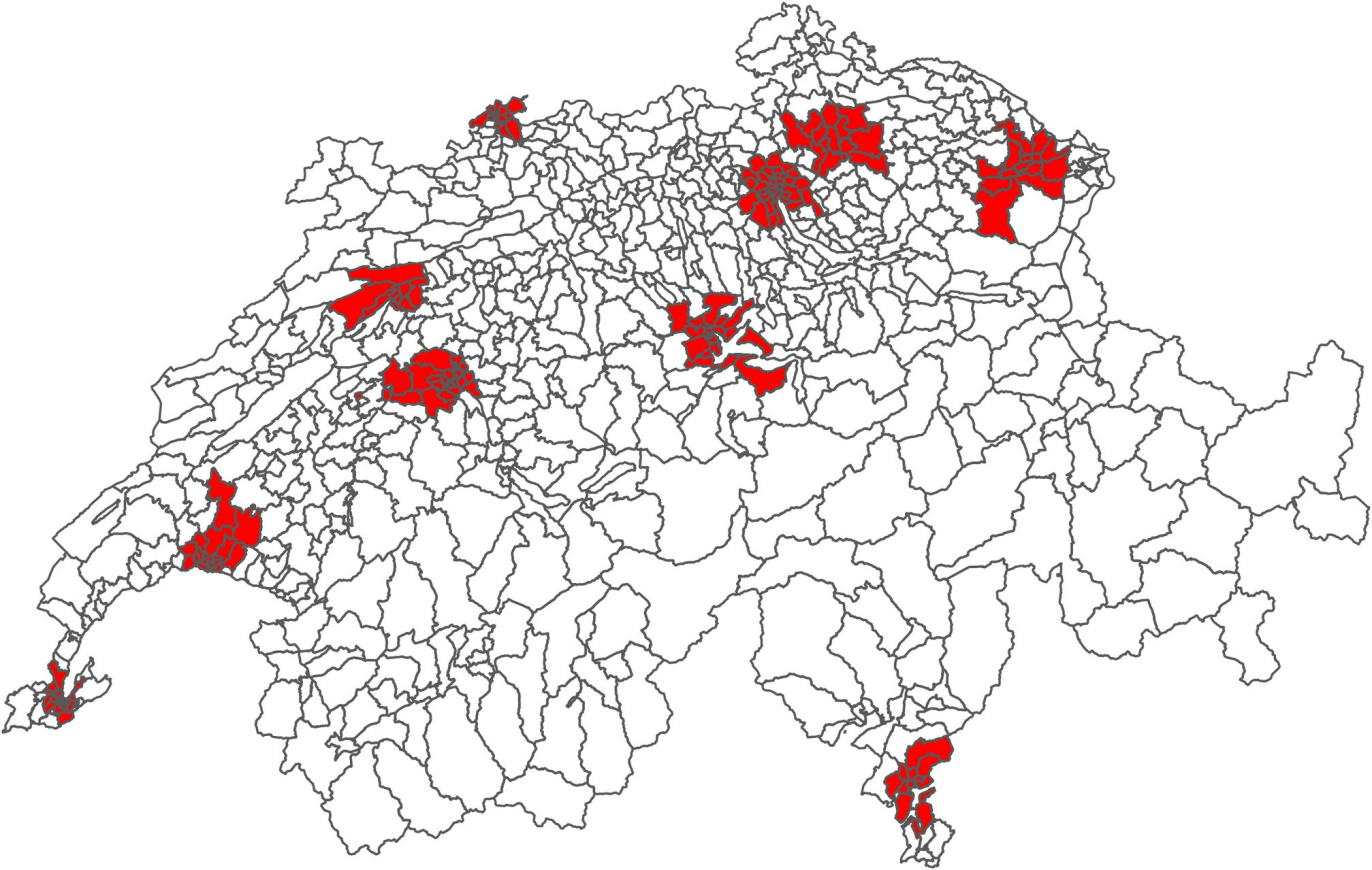
Assigned MedStat regions for each city.

Daily hospital admission data registered between 2009 and 2019 were obtained from Switzerland’s Federal Office of Statistics for the eight main Swiss cities. The cities covered in this study were located in regions of Switzerland that speak French (Geneva and Lausanne), German (Basel, Bern, Lucerne, St. Gallen, and Zurich), and Italian (Lugano). Swiss hospitals provided an anonymized and standardized data set, and diagnoses for mental disorders (F00—F98) were classified according to the International Classification of Diseases, 10th revision, German Modification (ICD-10-GM). The database provided the patient’s residence anonymity in 706 geographical units, so-called MedStat regions, a unit created by the aggregation of postal codes. Selected MedStat regions for this study were presented in the map in Fig 1. For this study, daily hospital admission data were aggregated by sex, age group (0–64 years and ≥ 65 years), and specific sub-diagnosis (refer to the list of diagnoses in Table 1). We used the same grouping for a subgroups of diagnosis from the previous study [16].

**Table 1:** Summary statistics of hospital admissions due to mental disorders in the eight main cities in Switzerland during 2009–2019.

		N	%
Total		147468	
Sex	Male	71891	48.8
	Female	75577	51.2
Age	< 65 years old	114722	77.8
	≥ 65 years old	32746	22.2
Diagnosis	Total	143713*	
	Organic, including symptomatic, mental disorders (F00-F09)	17029	11.8
	Mental and behavioral disorders due to psychoactive substance use (F10-F19)	33730	23.5
	Schizophrenia, schizotypal and delusional disorders (F20-29)	29645	20.6
	Mood disorders (F30-39)	30187	21.0
	Neurotic disorders (F40-59)	20765	14.4
	Disorders of adult personality and behavior (F60-69)	8614	6.0
	Mental retardation (F70-79)	1083	0.8
	Developmental disorders (F80-98)	2660	1.9

* Data on diagnosis for 3755 patients (2.5%) was not specified in IDC-10 code.

### Meteorological data

Daily meteorological data were obtained from the Federal Office of Meteorology and Climatology, MeteoSwiss. We used 2 km x 2 km daily accumulated precipitation data and daily mean temperature data with 1.6 km x 2.3 km resolutions. These datasets covered the whole of Switzerland for the period from 1961 to 2019. We derived daily city-level meteorological series by averaging the data from grid cells intersecting the selected MedStat regions in each of the eight main cities of Switzerland.

For sensitivity analysis, we used river discharge data obtained from the Federal Office for the Environment. The data used in this study was from January 2009 to December 2019 for each catchment. Lugano was excluded from the analysis since river discharge data was not available.

## Methods

### Identification of precipitation event based on the different definition

As proposed by Bulbena et al. [25] and Medici et al. [26], we derived different precipitation metrics based on the duration—persistent precipitation (PP)—and intensity—persistent extreme precipitation (PEP). This study defined PPs as the event when the daily precipitation was ≥ 1mm for 2, 3, or 4 days (PP.2, PP.3, PP.4). Also, Froidevaux et al. [7] showed that precipitation accumulations 2–3 days before were most relevant to the occurrence of flooding. Thus, PEP was defined as the event which exceeded the 90th percentile of the 30-year (1990–2019) baseline for 2 consecutive days (PEP90.2).

### Statistical analysis

**Fig 2:**
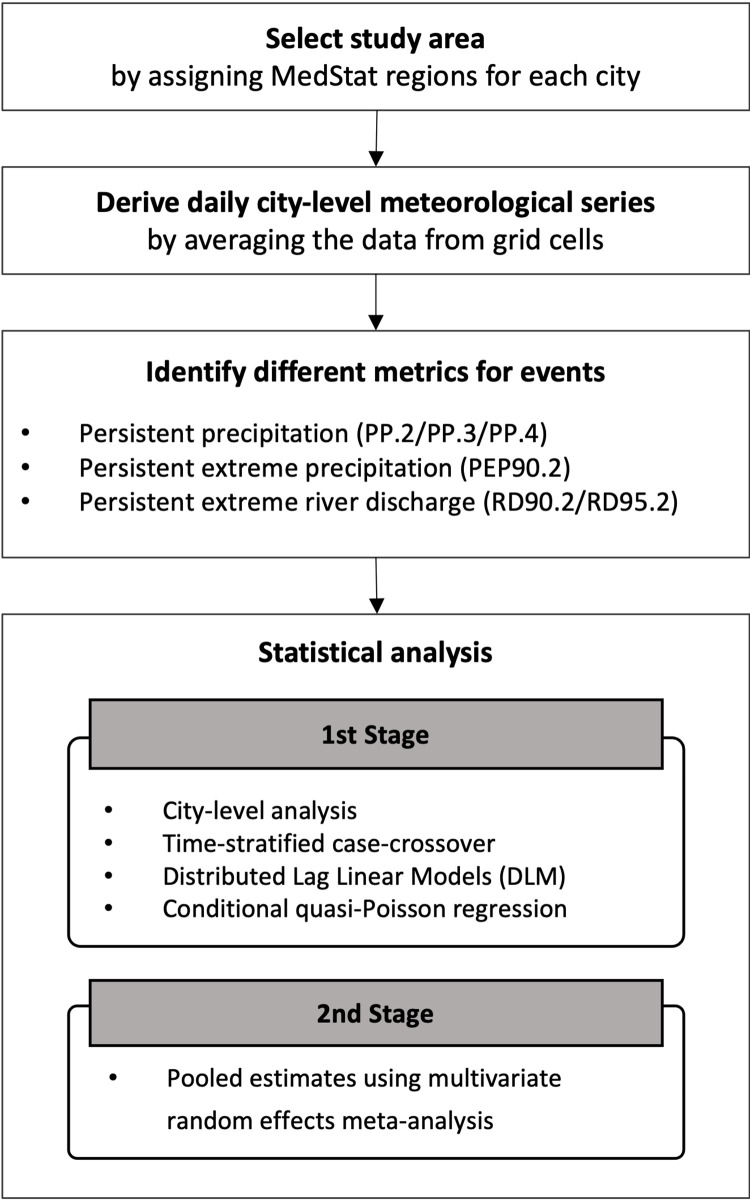
Diagram of research design and procedures.

We used a two-stage approach to assess the relationship between precipitation and hospital admissions for mental disorders (Fig 2). In brief, we first conducted aggregated time-stratified case-crossover analysis in each city. We combined this with a distributed lag linear model (DLM), which simultaneously assesses the response-exposure association and the lagged effect of environmental factors [36]. Second, we pooled the city-specific estimates to obtain the overall associations between precipitation and hospital admissions for mental disorders for total and by subgroups (sex, age, diagnosis) using a random effects meta-analysis.

First, we performed aggregated time-stratified case-crossover analysis in each of the eight selected cities in Switzerland. We performed conditional quasi-Poisson regression with DLM to allow for the overdispersion of the daily hospital admission counts and account for delayed associations [36,37]. The model used in this research was as follows:

$$Y_{t}∼quasi\;Poisson\;(\mu_{t})$$

$$\log\left(\mu_{t}\right)=\alpha_{stratumk}+cb({Prec}_{t,l})+cb({Temp}_{t,l})+\gamma DOW$$

where *μ*_*t*_ represented the number of daily hospital admission due to mental disorders on the day of observation *t*. *α*_*stratum k*_ was the intercept of stratum *k*. We used a time-stratified model with a stratum variable to control for long-term and seasonal trends by matching the case day with its control days according to the same month and the same year. *cb(Prec*_*t*,*l*_*)* was the cross-basis matrix of defined precipitation events on day *t* and lag *l* (in this case, 3 days) produced by DLM to estimate the delayed effects of precipitation. Here, a precipitation event was defined as a dummy variable (PP/PEP event and no event were represented as 1 and 0, respectively). Regarding the lag-response dimension, the delayed effects of precipitation were defined by two strata intervals at lag 0 and lag 1–3. *cb(Temp*_*t*,*l*_*)* is the cross-basis function of mean temperature based on the specification suggested by Bundo et al. [16], to adjust for the effect of daily mean temperature on hospital admission due to mental disorders. The effect of temperature was assumed as linear, and we modeled the lag-response dimension with an integer function with a lag of 3 days. *DOW* was the day of the week of hospital admission, which was also considered a potential confounder. The cross-basis specifications were selected according to quasi-Akaike (qAIC). We conducted statistical analysis for each definition of precipitation events (PP.2, PP.3, PP.4, and PEP90.2).

The city-specific estimates from the first stage were pooled in a random effects meta-regression model in the second stage to derive the overall risk. The main analysis was conducted for the total daily counts of hospital admissions from 2009 to 2019 and by subgroups of sex, age (0–64 and ≥ 65), and diagnosis in accordance with the ICD-10 categorization. We only reported overall estimates regarding the stratified analysis by sex, age, and specific causes. Additionally, seasonal analysis was conducted using the same model as the main analysis. We defined the warmer (May to October) and colder (November to April) seasons based on the temperature distribution. We tested for differences across categories of sex, age, sub-diagnoses, and season with a Wald-test performed in the meta-analysis model. Specifically, for each covariate, we combined the city-specific coefficients obtained in the stratified analysis in a meta-regression model with an indicator of the category (P-values under 0.05 were considered as statistically significant). Furthermore, we used Cochran’s Q test and the I^2^ statistic to assess the heterogeneity in the risk of hospital admission for mental disorders among cities.

We conducted separated statistical analyses using the RStudio program (version 4.0.2, R Core Team) (we specifically used the “dlnm”, “mixmeta”, and “gnm” packages).

### Sensitivity analysis

Previous research revealed that several successive days with heavy precipitation strongly increase flood risk [8,35,38–40]. Thus, we conducted a sensitivity analysis to see if there is a similarity between the results of persistent extreme precipitation and consecutive extreme discharge events. First, we identified persistent extreme river discharge (RD) events in the same ways as precipitation events: RD90.2/RD95.2 were defined as the event when daily river discharge data exceeded the 90th and 95th percentile for at least 2 consecutive days.

## Results

### Descriptive statistics

A total of 147,468 hospital admissions due to mental disorders were registered in the study area between 2009 and 2019. Among these, 51.2% (75,577) were females, and 77.8% (114,722) were under age 65 (Table 1). Mental and behavioral disorders due to psychoactive substance use (23.5%) were the most frequent sub-diagnosis, followed by mood disorders (21%) and schizophrenia (20.6%). As data on diagnoses for 3,755 patients (2.5%) were unspecified mental disorders according to the ICD-10 code, the data was excluded from the analysis for sub-diagnosis.

Table 2 summarizes the daily precipitation distribution for each of the eight cities during the study period. The daily accumulated precipitation ranged from 0 to 116.70 mm across the cities. While the maximum daily-mean precipitation was highest in Lugano (4.78 mm/day), Basel recorded the minimum daily-mean precipitation (2.3 mm/day). In the case of the number of events, Lugano has the highest number of PEP90.2 events (214), while Bern has the lowest number of PEP90.2 events (119) during the study period.

**Table 2:** Summary statistics of precipitation in eight cities of Switzerland between 2009 and 2019.

	Zurich	Bern	Basel	Geneva	Lugano	Lausanne	Luzern	St. Gallen
**Daily Precipitation (mm/day)**								
**Min**	0	0	0	0	0	0	0	0
**P25**	0	0	0	0	0	0	0	0
**P50**	0.1	0.05	0.05	0	0.01	0.03	0.2	0.23
**Mean**	2.99	2.71	2.3	2.35	4.78	2.95	3.54	3.7
**P75**	3.16	2.74	2.31	1.86	2.91	2.93	4.18	4.37
**P90**	9.58	9.19	7.6	8.18	15.45	10.14	11.67	11.72
**Max**	58.05	64.73	43.94	70.3	116.79	62.61	67.83	73.41
**Number of selected events (n)**									
**PP.2**	1194	1105	1020	924	1065	1114	1356	1364
**PP.3**	842	765	736	570	791	776	1004	1006
**PP.4**	192	152	220	132	196	172	260	244
**PEP90.2**	137	119	158	146	214	146	132	153

### Association between precipitation and hospital admissions due to mental disorders

There was no robust evidence of a consistent association between precipitation and hospital admissions for mental disorders in Switzerland between 2009 and 2019 (PP.2: 1.003 [0.978–1.029]; PP.3: 1.005 [0.985–1.026]; PP.4: 0.994 [0.960–1.030]; PEP90.2: 1.000 [0.953–1.050]). In the seasonal analysis, although highly uncertain, the risk of hospital admission seemed to increase with increasing intensity of precipitation in warmer seasons (PP.2: 1.001 [0.971–1.032] vs PEP90.2: 1.014 [0.955–1.078]). However, this pattern was opposite in the colder season, even though it was not robust, showing that the risks of hospital admission slightly increased by the duration (PP.2: 1.009 [0.981–1.039] vs PP.3: 1.008 [0.980–1.036] vs PP.4: 1.017 [0.956–1.081]) (Table 3).

**Table 3:** Pooled association estimates between hospital admissions due to mental disorders and PP.2 PP.3, PP.4, and PEP90.2 events with lag 3 (Relative Risk [95% confidence interval]).

	All	Season		Sex		Age	
		Warmer	Colder	Male	Female	< 65 yrs	≥ 65 yrs
**PP.2**	1.003 [0.978–1.029]	1.001 [0.971–1.032]	1.009 [0.981–1.039]	0.989 [0.961–1.017]	1.019 [0.990–1.049]	1.01 [0.984–1.036]	0.983 [0.944–1.024]
**PP.3**	1.005 [0.985–1.026]	1.007 [0.977–1.038]	1.008 [0.980–1.036]	0.99 [0.962–1.018]	1.020 [0.992–1.049]	1.011 [0.988–1.034]	0.985 [0.945–1.027]
**PP.4**	0.994 [0.960–1.030]	0.977 [0.929–1.027]	1.017 [0.956–1.081]	0.968 [0.920–1.018]	1.021 [0.972–1.073]	1.002 [0.962–1.043]	0.971 [0.902–1.044]
**PEP90.2**	1.000 [0.953–1.050]	1.014 [0.955–1.078]	0.972 [0.870–1.085]	1.025 [0.958–1.096]	0.974 [0.911–1.042]	1.004 [0.951–1.059]	0.984 [0.891–1.086]

* yrs: Years.

For subgroup analysis, although still uncertain, we observed that association estimates tended to be higher in the population aged < 65 years for both PPs, and PEP events compared to ≥ 65 years. In the analysis across the sex groups, although not robust, we found a positive association with PPs events (PP.2: 1.019 [0.990–1.049]; PP.3: 1.020 [0.992–1.049]; PP.4: 1.021 [0.972–1.073]) and a negative association with PEP90.2 events (0.974 [0.911–1.042]) in females. However, this pattern was the opposite in males, showing larger association estimates for PEP90.2 (1.025 [0.958–1.096]) compared to PPs events (Table 3).

**Fig 3:**
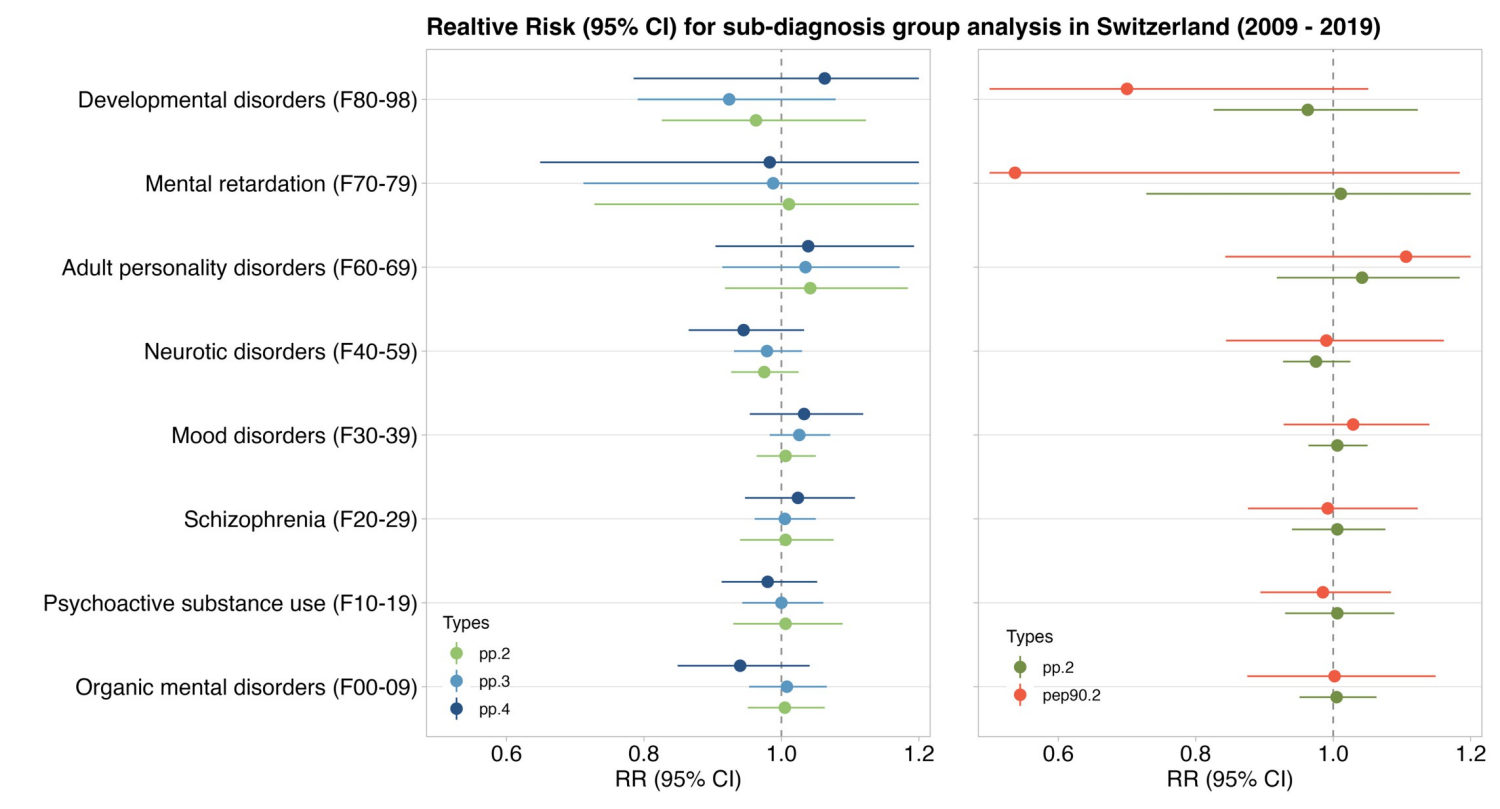
Pooled association estimates of sub-diagnosis group analysis between hospital admissions due to mental disorders and PP.2 PP.3, PP.4, and PEP90.2 events with lag 3 (Relative Risk [95% confidence interval]). It is represented in the perspective of duration (PP.2 vs PP.3 vs PP.4, left side) and the intensity (PP.2 vs PEP90.2, right side).

In the analysis for the sub-diagnosis group, positive associations although not robust were found in mood disorders (F30-39) and adult personality disorders (F60-69) for both PPs and PEP events (Fig 3). Schizophrenia (F20-29) showed an indication of positive association only with PPs events (Fig 3). However, neurotic disorders seemed to be negatively associated with PP.4 events and an almost null association in the rest of the diagnosis groups (Fig 3). Nevertheless, according to the results obtained in the Wald test, the overall risks of mental health-related hospital admissions were not significantly different across subgroups of sex, age, and diagnosis for all types of precipitation events (S1 Table). We also found that, although uncertain, the risk of schizophrenia (F20-29) was slightly higher in the colder period. In comparison, the risk of mood disorders (F30-39) and adult personality disorders (F60-69) was slightly higher in the warmer period (S2 Table). However, the overall risks of hospital admission for mental disorders were also not significantly different between warmer and colder period (S1 Table).

The city-specific analysis found an indication of a positive association but was highly uncertain between all definitions of precipitation events and hospital admissions for mental disorders in Bern and Basel (Table 4). In the case of Geneva, there was little indication of a positive association for PEP90.2 events (1.052 [0.937–1.182]), while it had a statistically not significant inverse association for PPs events (PP.2: 0.943 [0.897–0.991]; PP.3: 0.982 [0.931–1.036]; PP.4: 0.949 [0.860–1.047]). However, St. Gallen had the opposite pattern from Geneva showing the indication of positive association for PPs events (PP.2: 1.026 [0.959–1.097]; PP.3: 1.033 [0.968–1.102]; PP.4: 1.008 [0.903–1.125]), while the opposite pattern was observed for PEP90.2 events (0.954 [0.812–1.122]). In Lugano, a negative association between all definitions of events and hospital admissions for mental disorders was found (Table 4). Nevertheless, the differences in the overall risk of hospital admission among cities were not significant according to the results of Cochran’s Q test and the I^2^ statistic (S1 Table).

**Table 4:** Pooled association estimates of city-specific analysis between hospital admissions due to mental disorders and PP.2 PP.3, PP.4, and PEP90.2 events with lag 3 (Relative Risk [95% confidence interval]).

	PP.2	PP.3	PP.4	PEP90.2
**Zurich**	1.026 [0.988–1.065]	1.005 [0.967–1.044]	0.982 [0.917–1.052]	1.004 [0.913–1.104]
**Bern**	1.005 [0.944–1.071]	1.028 [0.964–1.095]	1.111 [0.981–1.259]	1.049 [0.895–1.231]
**Basel**	1.003 [0.957–1.051]	1.004 [0.957–1.053]	1.012 [0.937–1.092]	1.015 [0.912–1.131]
**Geneva**	0.943 [0.897–0.991]	0.982 [0.931–1.036]	0.949 [0.860–1.047]	1.052 [0.937–1.182]
**Lugano**	0.951 [0.865–1.046]	0.994 [0.903–1.094]	0.927 [0.780–1.102]	0.905 [0.749–1.095]
**Lausanne**	1.058 [0.984–1.137]	1.029 [0.957–1.106]	0.940 [0.818–1.080]	0.928 [0.779–1.105]
**Luzern**	1.011 [0.943–1.084]	0.979 [0.915–1.048]	1.020 [0.918–1.133]	1.000 [0.843–1.186]
**St. Gallen**	1.026 [0.959–1.097]	1.033 [0.968–1.102]	1.008 [0.903–1.125]	0.954 [0.812–1.122]

### Sensitivity analysis

The sensitivity analysis was carried out at the city scale. We found positive but not robust association estimates for persistent extreme discharge events in Bern (RD90.2: 1.085 [0.982–1.199]; RD95.2: 1.121 [0.983–1.278]) and Geneva (RD90.2: 1.082 [0.996–1.175]; RD95.2: 1.042 [0.931–1.166]). However, we could not find a consistent association between persistent extreme discharge events and hospital admissions due to mental disorders in the rest of the cities (S3 Table).

## Discussion

In this study, we analyzed the association between different definitions of precipitation events and hospital admissions due to mental disorders in the eight main cities of Switzerland between 2009 and 2019. Overall, we did not find evidence of an association between precipitation and hospital admissions due to mental disorders. However, we observed interesting patterns in the results of sub-group diagnosis with different effects depending on the season and precipitation event types. In the warmer period, we observe an indication of increasing risk with increasing the intensity of rainfall events. In comparison, the risk seems to rise with a longer duration in the colder period. This suggests that the duration of the rainfall events may have more impact on the risks of hospital admission in the colder season. In contrast, the intensity of the precipitation might pose a greater risk in the warmer season. However, we acknowledge the large imprecision of our estimates and that these patterns need to be confirmed in future studies with a larger sample size or longer study period.

Our findings suggest that patients in Bern and Geneva may be more vulnerable to persistent extreme precipitation and high river discharge events than in other cities. The heterogeneous population in Switzerland may be the reason why the association estimates vary among the eight major cities of Switzerland. Different urbanicity, language, and cultural profiles characterize each main city of Switzerland, and these heterogeneous population characteristics could result in varying levels of vulnerability between cities. Furthermore, Switzerland’s flood risk management systems differ from cantonal authorities [41]. Therefore, the differences in cantonal interventions might also contribute to the regional patterns. Another possible reason is the experience of patients with extreme precipitation events. For example, Lugano had the highest number of PEP90.2 events during the study period. Thus, patients living in Lugano have more experience with heavy precipitation events. This could create the ability to adapt and overcome the stress caused by extreme weather events, which could provide a protective impact on hospital admissions for mental disorders.

In the sub-group analysis, although our results were not significantly different across sex groups, these suggest that the precipitation intensity may pose a slightly greater risk in males, while the duration might increase the risk of hospital admissions in females. Our findings may be consistent with a previous study, which showed that the proportion of females having depressive symptoms peaked in moderate rain [42]. Several studies suggested the light sensitivity of females to the reproduction hormone axis as the possible mechanism for the differential effect between females and males [43,44]. A greater number of rainy days is often linked to reduced sunshine duration. Due to the different light sensitivity between males and females, less exposure to sunlight can cause a different reaction by increasing the risk of mental disorders in females. However, we could not test this hypothesis since data on sunshine duration was not consistently available across locations and along the whole study period.

Regarding schizophrenic disorders, our results found an indication of a positive association with PPs events, especially in the colder period. Several studies showed a positive association between precipitation or flood and hospital admission due to schizophrenia [24,28,45,46]. However, these studies did not differentiate the types of precipitation events, since these can have a different impact depending on the precipitation’s length and intensity. Another study also suggested that the risk of hospital admission for schizophrenia was more likely to be affected by cold temperatures than warm temperatures [17]. However, a previous study conducted in Bern, Switzerland, showed a positive association between schizophrenia and increasing ambient temperature [16]. This contradictory finding would suggest that not only high temperature, but also rainy environments during cold season can also be a possible reason for increasing the risk of hospital admissions for schizophrenia in Switzerland.

Patients seemed to be more affected by prolonged and extreme precipitation events in terms of hospital admissions due to mood disorders (F30 –F39) and adult personality disorders (F60 –F69). However, estimates were not significantly different across subgroups of diagnosis. Concerning mood disorders, several studies showed that the amount of precipitation is positively associated with increasing depressive symptoms [13,47]. Several studies suggested sunshine duration as a potential driver of mental health issues. Deng et al. [48] found an association between higher solar radiation and reduced emergency visits for mood disorders. Harb et al. [49] proved that less exposure to sunlight was related to increasing cortisol and decreasing melatonin levels, which were connected to depressive symptoms. Furthermore, Lieverse et al. [50] showed that Bright Light Treatment improved depression symptoms in the elderly. Abbasi [47] also suggested that the changing precipitation regimes could be a possible reason for the depression. However, as mentioned before, we could not adjust the impact of sunshine duration in our study due to data availability. Because increased daily precipitation may have an inverse effect on daily sunshine duration, future research needs to evaluate the joint effect of precipitation and sunshine duration on mental health. In addition to this, several studies suggested that altering trace element concentrations (Zn, Fe, Mn, Ca, and Mg) caused by changing precipitation patterns can be a biological mechanism to increase the risk of mental disorders [51–54].

Although statistically not significant, we found a negative association between neurotic disorders (F40 –F59) for all definitions of precipitation events. However, no consistent association was found in the rest of the subgroups of diagnosis. Although Deng et al. [48] found that rainfall hours had an immediate protective effect on mental disorders during the warmer season, there is still limited information on the biological mechanisms behind this effect. Future studies are required to examine the protective effects on mental disorders, including neurotic disorders.

Lastly, we acknowledge several limitations of this study. First, estimates cannot be seen as causal associations due to the ecological nature of the study. Additionally, there could be potential misclassification of exposure, although this would be reflected in terms of the uncertainty of the estimates. Second, due to the lack of data, we could not assess the potential differential effects by specific individual characteristics, such as medical history, socio-economic status, and cultural connectivity of patients. Thus, further studies are needed to investigate pathophysiological mechanisms that may link extreme precipitation events and negative mental health outcomes based on patients’ individual characteristics. Third, we did not account for potential confounders such as sunshine duration, air pollution, and humidity since data were not available at the same geographical unit and across the whole study period. Thus, we cannot discard that residual confounding might be present in our association estimates.

## Conclusions

To the best of our knowledge, this research is the first multi-city study evaluating the association between precipitation events and hospital admissions due to mental disorders in Switzerland and elsewhere.

Overall, we did not find evidence of an association between precipitation and hospital admissions for mental disorders in Switzerland between 2009 and 2019. However, our findings suggest that the risk pattern of hospital admissions due to mental disorders associated with precipitation events may differ between seasons and across sex, age groups, and sub-diagnosis. Further studies across other geographies and at a larger scale are warranted to clarify whether precipitation should be considered a risk factor for worsening mental health. This could be of particular relevance given current projections of more extreme precipitation events driven by climate change during the coming decades in Switzerland and elsewhere.

## Supporting information

S1 Fig The temporal evolution of hospital admissions due to mental disorders and monthly average precipitation across the study period (2009–2019) in eight major Swiss cities. (TIF)

S1 Table Significance levels obtained in A) the Wald test and B) Cochran Q-test to determine the implication of sex, age, diagnosis, season, and geographical unit (city) as explanatory factors of heterogeneity between results. (DOCX)

S2 Table Pooled association estimates of seasonal analysis between hospital admissions for mental disorders and PP.2 PP.3, PP.4, and PEP90.2 events with lag 3 (Relative Risk [95% confidence interval]). (DOCX)

S3 Table Pooled association estimates of sensitivity analysis between hospital admissions for mental disorders and consecutive extreme river discharge events with lag 3 (Relative Risk [95% confidence interval]). (DOCX)
