# Supplementary figures and images for: Exploring the association between precipitation and hospital admission for mental disorders in Switzerland between 2009 and 2019
Source: PLoS One. 2023 Apr 24;18(4):e0283200. doi: 10.1371/journal.pone.0283200 (PMC10124868; doi:10.1371/journal.pone.0283200)

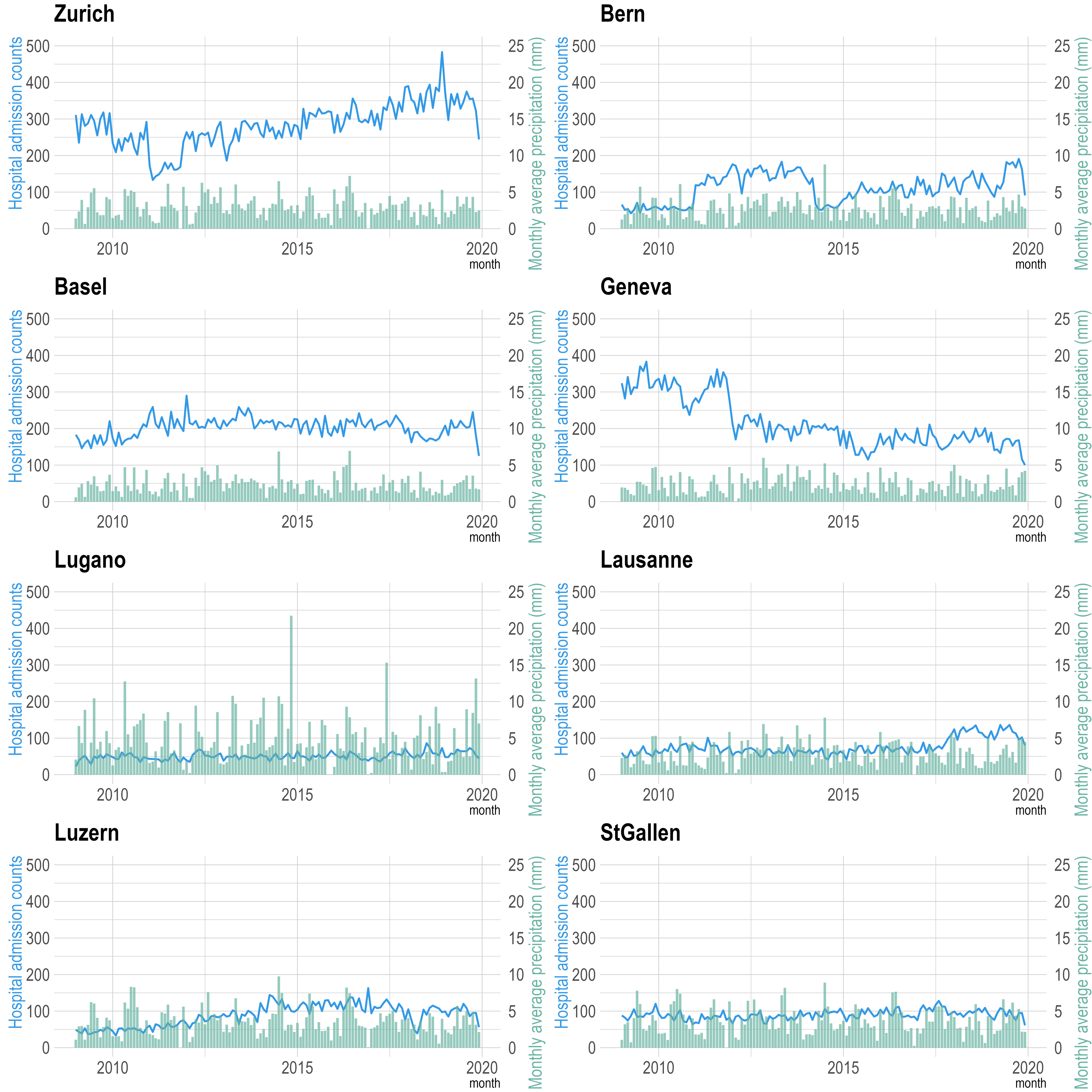

Supplement: S1 Fig — (TIF) [file pone.0283200.s001.tif]
