# Supplementary material for: Exploring the association between precipitation and hospital admission for mental disorders in Switzerland between 2009 and 2019
Source: PLoS One. 2023 Apr 24;18(4):e0283200. doi: 10.1371/journal.pone.0283200 (PMC10124868; doi:10.1371/journal.pone.0283200)
Supplement: S1 Table — (DOCX) [file pone.0283200.s002.docx]

**S1 Table. Significance levels obtained in A) the Wald test and B) Cochran Q-test to determine the implication of sex, age, diagnosis, season, and geographical unit (city) as explanatory factors of heterogeneity between results.**

**1) Wald test**

| **Types of precipitation events** | **Meta-predictors** | **Wald test (p-value)** |
| --- | --- | --- |
| PP.2 | Sex | 0.109 |
|  | Age | 0.359 |
|  | Diagnosis | 0.922 |
|  | Season | 0.719 |
| PP.3 | Sex | 0.120 |
|  | Age | 0.309 |
|  | Diagnosis | 0.765 |
|  | Season | 0.892 |
| PP.4 | Sex | 0.143 |
|  | Age | 0.582 |
|  | Diagnosis | 0.383 |
|  | Season | 0.222 |
| PEP 90.2 | Sex | 0.308 |
|  | Age | 0.709 |
|  | Diagnosis | 0.698 |
|  | Season | 0.514 |

**2) Cochran Q-test**

| **Types of precipitation events** | **Q** | **p-value** | **I^2^ statistics (%)** |
| --- | --- | --- | --- |
| PP.2 | 2.738 | 0.740 | 0.000 |
| PP.3 | 2.397 | 0.792 | 0.000 |
| PP.4 | 6.822 | 0.234 | 26.702 |
| PEP90.2 | 2.237 | 0.815 | 0.000 |
