# Supplementary material for: Exploring the association between precipitation and hospital admission for mental disorders in Switzerland between 2009 and 2019
Source: PLoS One. 2023 Apr 24;18(4):e0283200. doi: 10.1371/journal.pone.0283200 (PMC10124868; doi:10.1371/journal.pone.0283200)
Supplement: S2 Table — (DOCX) [file pone.0283200.s003.docx]

**S2 Table. Pooled association estimates of seasonal analysis between hospital admissions for mental disorders and PP.2 PP.3, PP.4, and PEP90.2 events with lag 3 (Relative Risk [95% confidence interval])**

**1) Warmer Period (May – October)**

|  | **PP.2** | **PP.3** | **PP.4** | **PEP90.2** |
| --- | --- | --- | --- | --- |
| **Total** | 1.001 [0.971 - 1.032] | 1.007 [0.977 - 1.038] | 0.977 [0.929 - 1.027] | 1.014 [0.955 - 1.078] |
| **0 – 64 yrs** | 1.009 [0.975 - 1.044] | 1.010 [0.976 - 1.045] | 0.986 [0.932 - 1.043] | 1.012 [0.945 - 1.084] |
| **65 yrs** | 0.974 [0.915 - 1.037] | 0.993 [0.933 - 1.057] | 0.941 [0.845 - 1.048] | 1.013 [0.893 - 1.149] |
| **Male** | 0.971 [0.931 - 1.013] | 0.984 [0.943 - 1.027] | 0.936 [0.872 - 1.005] | 1.047 [0.962 - 1.139] |
| **Female** | 1.030 [0.987 - 1.074] | 1.028 [0.986 - 1.073] | 1.015 [0.947 - 1.088] | 0.981 [0.901 - 1.068] |
| **F00-F09** | 1.011 [0.927 - 1.102] | 1.016 [0.916 - 1.127] | 0.905 [0.782 - 1.048] | 1.014 [0.850 - 1.210] |
| **F10-F19** | 1.007 [0.900 - 1.127] | 0.986 [0.881 - 1.104] | 0.937 [0.847 - 1.037] | 0.950 [0.841 - 1.074] |
| **F20-F29** | 1.012 [0.948 - 1.080] | 0.988 [0.925 - 1.055] | 0.966 [0.866 - 1.079] | 1.008 [0.874 - 1.163] |
| **F30-F39** | 0.985 [0.923 - 1.052] | 1.036 [0.971 - 1.105] | 1.041 [0.936 - 1.158] | 1.090 [0.958 - 1.240] |
| **F40-F59** | 0.977 [0.906 - 1.054] | 0.997 [0.925 - 1.076] | 0.909 [0.794 - 1.040] | 1.007 [0.838 - 1.210] |
| **F60-F69** | 1.096 [0.972 - 1.237] | 1.055 [0.932 - 1.194] | 1.131 [0.934 - 1.370] | 1.069 [0.789 - 1.449] |
| **F70-F79** | 0.913 [0.518 - 1.607] | 0.843 [0.490 - 1.452] | 0.896 [0.472 - 1.702] | 0.637 [0.188 - 2.165] |
| **F80-F98** | 1.096 [0.868 - 1.384] | 1.039 [0.804 - 1.343] | 1.121 [0.694 - 1.808] | 0.732 [0.345 - 1.555] |

* Remarks: There were no events of F70-F79 in Lausanne during the warmer period

**2) Colder Period (November – April)**

|  | **PP.2** | **PP.3** | **PP.4** | **PEP90.2** |
| --- | --- | --- | --- | --- |
| **Total** | 1.009 [0.981 - 1.039] | 1.008 [0.980 - 1.036] | 1.017 [0.956 - 1.081] | 0.972 [0.870 - 1.085] |
| **0 – 64 yrs** | 1.012 [0.980 - 1.044] | 1.012 [0.980 - 1.045] | 1.019 [0.930 - 1.118] | 0.950 [0.826 - 1.093] |
| **65 yrs** | 1.004 [0.950 - 1.062] | 0.993 [0.938 - 1.051] | 1.002 [0.903 - 1.113] | 0.991 [0.832 - 1.182] |
| **Male** | 1.007 [0.969 - 1.047] | 0.999 [0.960 - 1.039] | 1.015 [0.944 - 1.091] | 0.991 [0.877 - 1.119] |
| **Female** | 1.011 [0.973 - 1.050] | 1.015 [0.976 - 1.055] | 1.019 [0.938 - 1.106] | 0.949 [0.822 - 1.096] |
| **F00-F09** | 1.024 [0.949 - 1.105] | 1.026 [0.951 - 1.108] | 0.958 [0.827 - 1.109] | 1.058 [0.799 - 1.402] |
| **F10-F19** | 1.002 [0.948 - 1.059] | 1.009 [0.954 - 1.067] | 1.041 [0.939 - 1.153] | 0.984 [0.826 - 1.172] |
| **F20-F29** | 1.019 [0.926 - 1.121] | 1.016 [0.944 - 1.093] | 1.093 [0.976 - 1.224] | 0.999 [0.802 - 1.243] |
| **F30-F39** | 1.020 [0.961 - 1.082] | 1.017 [0.958 - 1.079] | 1.008 [0.902 - 1.127] | 0.880 [0.671 - 1.153] |
| **F40-F59** | 0.968 [0.904 - 1.037] | 0.959 [0.895 - 1.029] | 0.980 [0.819 - 1.173] | 0.917 [0.736 - 1.144] |
| **F60-F69** | 1.005 [0.887 - 1.138] | 1.023 [0.904 - 1.157] | 0.894 [0.696 - 1.149] | 1.264 [0.889 - 1.797] |
| **F70-F79** | 1.004 [0.730 - 1.382] | 1.077 [0.784 - 1.480] | 0.985 [0.537 - 1.809] | 0.953 [0.252 - 3.606] |
| **F80-F98** | 0.839 [0.678 - 1.038] | 0.805 [0.646 - 1.003] | 0.929 [0.533 - 1.621] | 1.255 [0.547 - 2.881] |
