# Supplementary material for: Exploring the association between precipitation and hospital admission for mental disorders in Switzerland between 2009 and 2019
Source: PLoS One. 2023 Apr 24;18(4):e0283200. doi: 10.1371/journal.pone.0283200 (PMC10124868; doi:10.1371/journal.pone.0283200)
Supplement: S3 Table — (DOCX) [file pone.0283200.s004.docx]

**S3 Table. Pooled association estimates of sensitivity analysis between hospital admissions for mental disorders and consecutive extreme river discharge events with lag 3 (Relative Risk [95% confidence interval]).**

|  | **RD90.2** | **RD95.2** |
| --- | --- | --- |
| **Zurich** | 0.964 [0.906 - 1.027] | 0.938 [0.866 - 1.016] |
| **Bern** | 1.085 [0.982 - 1.199] | 1.121 [0.983 - 1.278] |
| **Basel** | 0.978 [0.904 - 1.059] | 0.915 [0.823 - 1.018] |
| **Geneva** | 1.082 [0.996 - 1.175] | 1.042 [0.931 - 1.166] |
| **Lugano** | - | - |
| **Lausanne** | 0.919 [0.822 - 1.027] | 0.829 [0.703 - 0.978] |
| **Luzern** | 0.922 [0.816 - 1.041] | 0.885 [0.765 - 1.023] |
| **St. Gallen** | 0.919 [0.822 - 1.026] | 0.917 [0.784 - 1.072] |

* Remarks: River discharge data was not available for Lugano
